# Concordance between 8-1-1 HealthLink BC Emergency iDoctor-in-assistance (HEiDi) virtual physician advice and subsequent health service utilization for callers to a nurse-managed provincial health information telephone service

**DOI:** 10.1186/s12913-023-09821-w

**Published:** 2023-09-27

**Authors:** Ross Duncan, Kurtis Stewart, Frank X. Scheuermeyer, Riyad B. Abu-Laban, Kendall Ho, Danielle Lavallee, Jim Christenson, Nancy Wood, Stirling Bryan, Lindsay Hedden

**Affiliations:** 1 Michael Smith Health Research British Columbia, Vancouver, Canada; 2 BC Emergency Medicine Network, Vancouver, Canada; 3 Department of Emergency Medicine, Faculty of Medicine, University of British Columbia, Vancouver, Canada; 4 School of Population and Public Health, Faculty of Medicine, University of British Columbia, Vancouver, Canada; 5 Faculty of Health Sciences, Simon Fraser University, Burnaby, Canada

**Keywords:** Telephone Triage and Advice (TTA), Emergency Medicine, Emergency Care, Primary Care, Health Services Utilization, Virtual Physicians, Physician Advice

## Abstract

**Background:**

British Columbia 8–1-1 callers who are advised by a nurse to seek urgent medical care can be referred to virtual physicians (VPs) for supplemental assessment and advice. Prior research indicates callers’ subsequent health service use may diverge from VP advice. We sought to 1) estimate concordance between VP advice and subsequent health service use, and 2) identify factors associated with concordance to understand potential drivers of discordant cases.

**Methods:**

We linked relevant provincial administrative databases to obtain inpatient, outpatient, and emergency service use by callers. We developed operational definitions of concordance collaboratively with researcher, patient, VP, and management perspectives. We used Kaplan–Meier curves to describe health service use post-VP consultation and Cox regression to estimate the association of caller factors (rurality, demography, attachment to primary care) and call factors (reason, triage level, time of day) with concordance as hazard ratios.

**Results:**

We analyzed 17,188 calls from November 16, 2020 to April 30, 2021. Callers advised to attend an emergency department (ED) immediately were the most concordant (73%) while concordance was lowest for those advised to seek Family Physician (FP) care either immediately (41%) or within 7 days (47%). Callers unattached to FPs were less likely to schedule an FP visit (hazard ratio = 0.76 [95%CI: 0.68–0.85]). Rural callers were less likely to attend an ED within 48 h when advised to go immediately (0.53 [95%CI:0.46–0.61]) compared to urban callers. Rural callers advised to see an FP, either immediately (1.28 [95%CI:1.01–1.62]) or within 7 days (1.23 [95%CI: 1.11–1.37]), were more likely to do so than urban callers.

**Interpretation:**

Concordance between VP advice and subsequent caller health service use varies substantially by category of advice and caller rurality. Concordance with advice to “Go to ED” is high overall but to access primary care is below 50%, suggesting potential issues with timely access to FP care. Future research from a patient/caller centered perspective may reveal additional barriers and facilitators to concordance.

**Supplementary Information:**

The online version contains supplementary material available at 10.1186/s12913-023-09821-w.

## Background

Many Canadian provinces have publicly funded, nurse-managed telephone services to support citizens with urgent health concerns. These services provide information on management and on whether, when, and where to seek appropriate care [1–5]. In British Columbia (BC), the nurse-managed telephone service involves an 8–1-1 phone line overseen by HealthLink BC (HLBC). The COVID-19 pandemic greatly increased both the volume and complexity of calls to 8–1-1, with many callers seeking to avoid the potential infection risk associated with in-person healthcare visits. This prompted HLBC to add a virtual physician (VP) consultation service, called HLBC Emergency iDoctors in-assistance (HEiDi) [6]. Through the HEiDi service, callers identified by 8–1-1 nurses as requiring medical attention immediately or as soon as feasible can be referred for supplemental virtual assessment by telephone or video by off-site emergency physicians, with the potential to safely divert some callers away from emergency departments (EDs).

### Nurse-led telephone triage and advice

Existing research suggests that concordance between advice given on nurse-managed telephone triage services and subsequent health service use ranges between 62 and 75%, [7, 8] with concordance with advice to see a family physician (FP) being particularly low [8]. A 2010 study of a nurse-led 8–1-1 line in Calgary, Alberta, found high “follow-through” for individuals advised to monitor symptoms at home, but relatively low “follow-through” on advice to seek care in an ED immediately (52.3%) or to seek care in the community within 24-h (43.2%) [9]. “Follow-through” also varied significantly by health concern [9]. A systematic review by Veteran’s Affairs found little evidence that nurse-led remote triage reduced downstream service use [10]. However, a more recent study found that patients who undergo initial remote triage using a validated algorithm are less likely to be given a low urgency triage when presenting to an ED [11], suggesting improvement in service provision.

### The context of ED and FP care and potential drivers of concordance

When examining concordance, it is important to consider trends documented in the literature around the use of health services. Of particular interest are patterns around the “appropriate” use of emergency care among subpopulations we may see reflected in BC. Lower levels of education have been positively associated with likelihood of both high and low acuity visits, with income showing a similar pattern [12]. Persons with a regular medical doctor have been found to be less likely to attend EDs [12]. Those living in a rural area have been found to be more likely to do so, and but less likely to present with high acuity [12]. Finally, a 2021 study found that patients living in areas with high scores for “deprivation” (Area Deprivation Index or ADI) or “stress” (neighborhood stress score or NSS) have a higher frequency of ED utilization [13].

A key concern for triage is that the ED often serves as a “safety net” for patients who lack access to appropriate primary care [14]. Lavergne et al.’s 2022 analysis of primary care service use and neighbourhood income in BC also found evidence of inequity in access to care [15]. From 1999/2000 through 2017/18, the number of primary care encounters declined in both urban and rural settings while ED visits increased [15]. This raises concerns for equity generally, but particularly for Indigenous patients, as those living in urban areas were found to have less access to primary care and consistently higher “preventable hospital admissions”, with an age-standardized mortality rate 2–5 times higher compared to other residents that authors felt was likely attributable to “gaps in access to primary care services.” [14].

### Physician assistance in telephone triage and advice

Research on the impact of the addition of VP consultation to nurse-managed telephone triage is extremely limited as few jurisdictions offer this service. Li et al. have published an evaluation of a similar program for veterans in the US which compares the clinical outcomes of telephone triage utilizing emergency physicians in addition to nurses versus nurses alone. However, the focus of this program was to better direct patients to in-network emergency departments rather than identify the most appropriate modality of care, precluding direct comparison to HEiDi [16]. In a previously published description and process evaluation of the HEiDi service, VPs advised 72.1% of urgently triaged callers away from visiting EDs or urgent care clinics within 24 h, while advising 15.0% of callers whose concerns were deemed less urgent by the nurse to attend an ED immediately [6]. Concordance between the VP’s recommendations and subsequent health service use has not been explored. Examining concordance with advice is an important step in understanding both the potential value of including VPs in nurse-led telephone services, and what shapes patients’ abilities and decisions to seek recommended follow-up care.

## Objective

This exploratory, hypothesis-generating analysis uses population-based administrative data to examine concordance between virtual physician advice and subsequent health services use.

We sought to 1) estimate concordance between VP advice and subsequent health service use, and 2) identify factors associated with concordance to understand potential drivers of discordant cases.

## Methods

### Study design

We conducted a retrospective, time-to-event (survival) analysis of routinely collected administrative health data. This study was approved by the University of British Columbia’s Clinical Research Ethics Board (H21-02209).

The study was conducted in British Columbia, Canada. As of April 2021, BC had approximately 5.2 million residents [17] distributed across five health administration regions (locally referred to as “health authorities” or HAs) [18] with a total of 120 hospitals [19] and 103 emergency departments [20].

### Data sources, linkage, and variables

We used BC Ministry of Health’s Health Data Platform BC (HDPBC) to access anonymized, linked administrative health data in a secure environment. Linkage between datasets was conducted using anonymized Personal Health Numbers (PHN).

HealthLink BC provided access to their 8–1-1 dataset to identify HEiDi callers during the study period. 8–1-1 data also included the level of nurse triage (“red-MD” or “yellow” indicating high and moderate urgency, respectively), health concern associated with call (e.g., “cardiovascular”, “pediatric”, “pharmaceutical (medications)”), time of call, and VP category of advice given (“disposition” – e.g. “Go To ED Now”). To mitigate potential confounding of concordance from multiple calls for the same or new issues, only the first call from each caller in a two-week period was used for this analysis. We used “cardiovascular” as the reference health concern for VP advice as this is widely understood as an urgent concern by both clinicians and the lay public. The most urban HA in BC, Vancouver Coastal, was used as the baseline for regional comparison. Finally, the 21–40 year-old age category was the most frequent and so used as the reference for comparisons across age.

We used the Discharge Abstract Database (DAD) [21] and National Ambulatory Care Reporting System (NACRS) [22] datasets to identify inpatient hospitalizations and ED visits up to seven days after the HEiDi call, and Medical Services Plan (MSP) billing information [23] to track use of outpatient services, particularly FP visits in the community for that same period. Both in-patient and remote (virtual/telehealth) services were tracked in MSP billings. This dataset also included a measure of “FP attachment”, based on a Ministry of Health algorithm that estimates whether a person has a consistent FP [24]. For the purposes of this analysis FP refers to practitioners identified by a speciality code of “00″, “Family Medicine”, for purposes of MSP billing [23]. Finally, demographic information (age, sex, rurality as per Statistics Canada’s definitions [25], and socioeconomics (SES) status via the "Canadian Index of Multiple Deprivation” [26] on callers was linked to the above using the “Client Roster” [27], and mortality was tracked by linking callers’ to Vital Events Deaths database [28].

The NACRS dataset, only includes data for approximately 29 EDs, typically at larger, urban hospitals constituting 70% of BC ED visits [29]. We therefore supplemented these data with billings from the MSP and DAD databases, following a previously described algorithm to improve ED visit ascertainment [30].

### HEiDi virtual physician advice and 8–1-1 “Disposition codes”:

We used consensus-derived operational definitions of existing HEiDi VP advice categories assigned by VPs at the end of each call, and the interpretation in Table 1 to describe the advice VPs gave to callers regarding seeking follow-up care.

**Table 1:** Operational definitions of VP advice “dispositions” and their interpretation

8–1-1 Disposition Code (software)	VP advice (Operational Definition)	Interpretation
Go to ED Now	Go to ED Now	Caller should proceed to an ED immediately
See Primary Care Now	Seek FP care now	The caller’s concern should be assessed by a FP as soon as possible
Schedule an MD/Healthcare Provider Appointment	Schedule FP appointment	The caller’s concern should be assessed in person in a timely manner
Try Home Treatment	Self-Manage	The caller does not have any immediate critical/urgent care needs and can self-manage and monitor their condition for any changes

### Study population

We have previously reported details of the 8–1-1 and HEiDi service and call-flow [6]. All of the “red-MD” or “yellow” callers interviewed by 8–1-1 nurses and referred for VP consultation between November 16, 2020 and April 30, 2021 – representing approximately 26% and 38% of 8–1-1 calls respectively – were included this analysis. We excluded any individuals with missing demographic data or whose PHN corresponded to a deceased individual at the time of call. We also excluded callers who received the “Other” disposition from the HEiDi physician, as this was insufficiently specific to allow assessment of concordance.

### Concordance

We used the following definitions of concordance, developed through consultation with HEiDi virtual physicians, health service researchers and a patient partner from the BC Emergency Medicine Network’s Patient Council. Virtual physicians strongly indicated that concordance could only reliably be assessed over a one-week (seven day) time-horizon, as over extended periods the likelihood that service use stems from issues unrelated to the reason for the index call increases (Table 2).

**Table 2:** Operational definitions of VP advice and corresponding concordance criteria

VP Advice	Concordance Criteria
Go to ED Now	The caller has a recorded ED visit within 48 h of the HEiDi call and did not have a recorded hospitalization or FP encounter before the ED visit
Seek FP care now^a^	The caller has a recorded FP encounter within seven days of the HEiDi call and did not have a recorded hospitalization or ED visit before the FP encounter
Schedule FP appointment^a^	The caller has a recorded FP encounter within seven days of the HEiDi call and did not have a recorded hospitalization or ED visit before the FP encounter
Self-Manage^b^	The caller has no recorded ED visits, hospitalizations, or FP encounters within seven days of the HEiDi call

^a^In conversations with VPs, they indicated that “seek FP care now” was considered more urgent than “schedule FP appointment” but noted that time to access FPs varies. A consensus existed that the appointment should be as soon as possible in the former scenario, and ideally within a week for the latter. Given potential access issues that may affect a plurality of patients, we included both dispositions with a one-week timeframe to assess if concordance patterns demonstrate a similar perception of urgency based on subsequent service use

^b^Note the final category advises that service use is unnecessary, and so “time-to-concordance” is inverted as “time-to-discordance.”

Concordance is considered achieved if the first service used by the caller subsequent to the call aligns with physician advice. Use of other services after this initial encounter is still considered concordant, but use of other services prior to the advised service does not. For example, for “Go to ED Now”: a caller who saw an FP or was hospitalized prior to their ED visit would not be counted as concordant. However, a caller who saw an FP and/or was hospitalized following their ED visit would be.

### Data analysis

We conducted a time-to-event (TTE) analysis using a Cox proportional hazard model [31] for each HEiDi disposition. Each of the models included the same set of independent variables, described in “Data Sources, Linkage and Variables” above. This was a hypothesis-generating analysis to determine the hazard ratios for each independent variable and for each disposition (as opposed to optimizing a predictive model with fewer predictors). A TTE analysis allowed us to estimate the effect of multiple predictors over the range of hours or days for each disposition.

We used SQL Server Management Studio (version 18.5, Microsoft) to query and link records across databases. Eligible records were extracted and imported to R (version 4.1, R Core Team) for statistical analysis.

### Sensitivity analysis

We also conducted sensitivity analysis with expanded definitions of concordance, incorporating broader health services use, for the three dispositions (Table 3).

**Table 3:** Operational Definitions of VP advice and corresponding sensitivity criteria for concordance

VP Advice	Sensitivity Testing Concordance Criteria
Go to ED Now	The caller has a recorded ED visit, FP encounter, specialist encounter, or laboratory/radiology test within 48 h of the HEiDi call and did not have a recorded hospitalization before the other encounters
Seek FP care now^a^	The caller has a recorded ED visit, FP encounter, specialist encounter, or laboratory/radiology test within seven days of the HEiDi call and did not have a recorded hospitalization before the other encounters
Schedule FP appointment^a^	The caller has a recorded ED visit, FP encounter, specialist encounter, or laboratory/radiology test within seven days of the HEiDi call and did not have a recorded hospitalization before the other encounters
Self-Manage^b^	No appropriate test available

^a^In conversations with VPs, they indicated that “seek FP care now” was considered more urgent than “schedule FP appointment” but noted that time to access FPs varies. A consensus existed that the appointment should be as soon as possible in the former scenario, and ideally within a week for the latter. Given potential access issues that may affect a plurality of patients, we included both dispositions with a one-week timeframe to assess if concordance patterns demonstrate a similar perception of urgency based on subsequent service use

^b^Note the final category advises that service use is unnecessary, and so “time-to-concordance” is inverted as “time-to-discordance.”

VPs expressed concern that strictly “binary” definitions of concordance may be misleading as advice given to callers often includes conditional statements and may be layered “You should do X unless Y changes, then look into doing Z”. This means the patient-caller could be initially told to “Seek FP care now” unless a given symptom worsens, in which case “Go to ED now”. We can only observe the initial layer of advice, but this sensitivity analysis allows us to view patterns of callers’ behaviour with the allowance for conditional advice unobservable in the data.

In this exploratory analysis, we examined sensitivity analysis results for any significant, systematic difference from primary analyses.

## Results

From November 16, 2020 through April 30, 2021, the HEiDi service recorded 19,186 VP encounters with 8–1-1 callers. 1998 calls (10.4%) were excluded: 946 (4.9%) due to missing data, 24 (0.1%) corresponding to a deceased individual, 253 (1.3%) for being a repeat call within 14 days. The callers whose disposition was labeled as “Other” (*n* = 775, 4.0%) were also excluded, leaving 17,188 calls included in the analysis.

### Caller and call characteristics

Most callers were women (64.3%) over age 21, with age 21–40 being the most frequent age group and median age of callers ranging from 35.6 to 42.1 years by disposition. Callers were disproportionately high SES as compared to the general population reported in BC census data, with 25.1% in the top quintile. Rurality varied slightly by disposition but was generally slightly higher than from provincial population estimates of ~ 13% [32]. Approximately 84% of callers were “attached” to a FP (Table 4).

**Table 4:** Summary of HEiDi caller and call characteristics by disposition

	**HEiDi Disposition**				
	**Go to ED Now** **(*n* = 2876, 16.7%)**	**See FP Now** **(*n* = 1512, 8.8%)**	**Schedule FP Appointment** **(*n* = 6649, 38.7%)**	**Self-Manage (*n* = 6151, 35.8%)**	**Total** **(*n* = 17,188)**
Number of unique callers	2871	1510	6589	6115	16,891
Mean (SD) HEiDi encounters per caller	1.00 (0.04)	1.00 (0.04)	1.01 (0.10)	1.01 (0.08)	1.02 (0.14)
8–1-1 nurse triage					
*Yellow*	1362 (47.4%)	981 (64.9%)	4944 (74.4%)	4409 (71.7%)	11,696 (68.0%)
*Red-MD*	1514 (52.6%)	531 (35.1%)	1705 (25.6%)	1742 (28.3%)	5492 (32.0%)
Time of call					
*Day*	1724 (59.9%)	1173 (77.6%)	3659 (55.0%)	3647 (59.3%)	10,203 (59.4%)
*Evening*	1036 (36.0%)	250 (16.5%)	2798 (42.1%)	2249 (36.6%)	6333 (36.8%)
*Off-hours*	116 (4.0%)	89 (5.9%)	192 (2.9%)	255 (4.1%)	652 (3.8%)
Weekday	1951 (67.8%)	1072 (70.9%)	4614 (69.4%)	4249 (69.1%)	11,886 (69.2%)
Health concern					
*Gastroenterology (digestive)*	583 (20.3%)	208 (13.8%)	1078 (16.2%)	1012 (16.5%)	2881 (16.8%)
*Musculoskeletal (bone, muscle, joint)*	376 (13.1%)	241 (15.9%)	894 (13.4%)	802 (13.0%)	2313 (13.5%)
*Neurology*	362 (12.6%)	160 (10.6%)	684 (10.3%)	683 (11.1%)	1889 (11.0%)
*Dermatology (skin, hair, nails)*	158 (5.5%)	147 (9.7%)	637 (9.6%)	719 (11.7%)	1661 (9.7%)
*Respiratory*	304 (10.6%)	140 (9.3%)	604 (9.1%)	569 (9.3%)	1617 (9.4%)
*Other*^*1*^	1093 (38.0%)	616 (40.7%)	2752 (41.4%)	2366 (38.5%)	6827 (39.7%)
Sex, female	1826 (63.5%)	1004 (66.4%)	4346 (65.4%)	3884 (63.0%)	11,050 (64.3%)
Age (years), mean (SD)	42.1 (25.3)	39.2 (23.6)	38.8 (24.2)	35.6 (24.3)	38.2 (24.5)
*0–1 years*	194 (6.7%)	93 (6.2%)	546 (8.2%)	670 (10.9%)	1503 (8.7%)
*2–5 years*	154 (5.4%)	86 (5.7%)	350 (5.3%)	442 (7.2%)	1032 (6.0%)
*6–20 years*	237 (8.2%)	137 (9.1%)	556 (8.4%)	569 (9.3%)	1499 (8.7%)
*21–40 years*	863 (30.0%)	533 (35.3%)	2315 (34.8%)	2013 (32.7%)	5724 (33.3%)
*41–65 years*	790 (27.5%)	422 (27.9%)	1779 (26.8%)	1618 (26.3%)	4609 (26.8%)
> *65 years*	638 (22.2%)	241 (15.9%)	1103 (16.6%)	839 (13.6%)	2821 (16.4%)
Attached to FP or practice	2441 (84.9%)	1255 (83.0%)	5578 (83.9%)	5176 (84.1%)	14,450 (84.1%)
Economic dependency, quintile					
*1: least deprived*	723 (25.1%)	411 (27.2%)	1751 (26.3%)	1687 (27.4%)	4572 (26.6%)
*2*	584 (20.3%)	315 (20.8%)	1375 (20.7%)	1307 (21.2%)	3581 (20.8%)
*3*	495 (17.2%)	279 (18.5%)	1245 (18.7%)	1148 (18.7%)	3167 (18.4%)
*4*	508 (17.7%)	269 (17.8%)	1169 (17.6%)	1048 (17.0%)	2994 (17.4%)
*5: most deprived*	566 (19.7%)	238 (15.7%)	1109 (16.7%)	961 (15.6%)	2874 (16.7%)
Health Authority					
*Interior*	507 (17.6%)	211 (14.0%)	1071 (16.1%)	937 (15.2%)	2726 (15.9%)
*Fraser*	1065 (37.0%)	541 (35.8%)	2391 (36.0%)	2522 (41.0%)	6519 (37.9%)
*Vancouver Coastal*	610 (21.2%)	347 (22.9%)	1458 (21.9%)	1375 (22.4%)	3790 (22.1%)
*Vancouver Island*	525 (18.3%)	344 (22.8%)	1369 (20.6%)	1042 (16.9%)	3280 (19.1%)
*Northern*	169 (5.9%)	69 (4.6%)	360 (5.4%)	275 (4.5%)	873 (5.1%)
Rural location	491 (17.1%)	203 (13.4%)	1010 (15.2%)	886 (14.4%)	2590 (15.1%)

Note: cell values represent count (percentage) or mean (standard deviation) as appropriate

^1^The “Other” health concern collapses the remaining 18 concerns, with the five most common health concerns displayed in this table. For a full list of health concerns, see Supplemental Table S1

Seventeen percent of calls triaged to VPs were advised to seek immediate emergency care, 48% were advised to seek care in the community, and 36% were advised to self-manage (Table 4) Most calls were placed during daytime business hours (69.2%) on a weekday (~ 70%). These characteristics have been evaluated in previously published work [24].

### Post-HEiDi health service utilizations

Callers assigned “Go to ED Now” were the most concordant at seven days (72.9%). Advice to “See FP now” was 49.6% and “Schedule FP Appointment” was 50.1%. Similarly, 48.9% of callers advised to “Self-Manage” did not use any additional services (Table 5). The “ED now” group was also 4 times more likely to be hospitalized than the others (8% vs 2%), suggesting high concern was justified. Specialist encounters and diagnostic testing appeared associated with the urgency of VP advice, with higher levels of concern seeing greater service use. FP visits were only slightly less frequent for “Go to ED Now” callers than FP-specific dispositions (45% vs 50%), and about 1/3 of those assigned to self manage saw an FP by week’s end (Table 5).

**Table 5:** Summary of health service utilizations within seven days by disposition following callers’ HEiDi encounter

	**HEiDi Disposition**			
	**Go to ED Now** **(*n* = 2876, 16.7%)**	**Seek FP care Now** **(*n* = 1512, 8.8%)**	**Schedule FP Appointment** **(*n* = 6649, 38.7%)**	**Self-Manage (*n* = 6151, 35.8%)**
**ED visits**				
*Number (%) of calls followed by at least 1 ED visit*	2098 (72.9%)	436 (28.8%)	964 (14.5%)	760 (12.4%)
**Hospitalizations**				
*Number (%) of calls followed by at least 1 hospitalization*	238 (8.3%)	31 (2.1%)	106 (1.6%)	126 (2.0%)
**FP encounters**				
*Number (%) of calls followed by at least 1 FP encounter*	1285 (44.7%)	750 (49.6%)	3329 (50.1%)	2081 (33.8%)
**Specialist encounters**				
*Number (%) of calls followed by at least 1 specialist encounter*	872 (30.3%)	326 (21.6%)	1139 (17.1%)	772 (12.6%)
**Laboratory/radiology tests**				
*Number (%) of calls followed by at least 1 test encounter*	963 (33.5%)	502 (33.2%)	2027 (30.5%)	1117 (18.2%)
**No health service utilizations**				
*Number (%) of calls followed by zero utilizations*	244 (8.5%)	328 (21.7%)	1939 (29.2%)	3008 (48.9%)

Concordance numbers can be seen at the intersection of advice and service use (ie. Go to ED Now – ED Visits, FP Encounters – See FP Now/Schedule FP Appointment, Self-Manage – No Health Service Utilization

### Time-to-event results

**Fig. 1:**
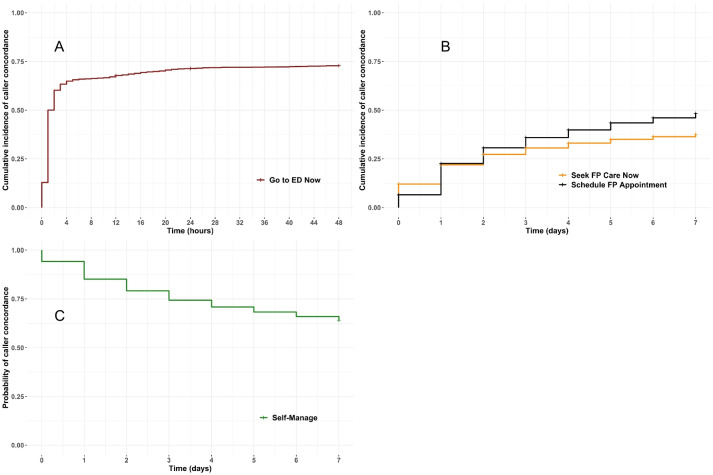
Adjusted Kaplan–Meier Curves for each category of VP Advice

As with absolute values, the rate of concordance varied across the four dispositions (Fig. 1, see Additional File 1: Table S2). A key finding evident in Fig. 1 is that concordance was highest “Go to ED Now”, where 70.4% of callers were found to have an ED visit within 48 h. We note this advice appears to have been acted on quickly—over 90% (1840/2025) of the subset of concordant callers visit an ED within the first 4 h (see Additional File 1: Table S2). Concordance was lowest for those told to seek FP care now or within seven days at 41% and 47% respectively. Sixty percent of callers in the “Self-Manage” disposition did not have an identified ED visit, hospitalization, or FP encounter within seven days.

While several geographic signals emerged from this analysis, only the Northern Health Authority was significantly associated with concordance across all four dispositions (Table 6). For the “Go to ED Now” disposition, HEiDi callers from Northern were less likely (hazard ratio = 0.56 [95%CI: 0.35–0.61]) while Fraser (1.16 [1.03–1.30]) and Island (1.39 [1.21–1.59]) were more likely to be concordant as compared to Vancouver Coastal. Rurally-located callers were significantly less likely to be concordant with advice to “Go to ED Now” (0.53 [0.46–0.61]) and those advised to see a family physician either immediately (1.28 [1.01–1.62]) or soon (1.23 [1.11–1.37]) were significantly more likely to record an FP encounter within seven days than urban callers.

**Table 6:** Summary of caller and call characteristics impact on concordance by category of advice (adjusted hazard ratios of multivariate cox regression)

Variable	Go to ED Now (n_events_ = 2025)	Seek FP Care Now (n_events_ = 625)	Schedule FP Appointment (n_events_ = 3091)	Self-Manage (n_events_ = 2471)
Nurse disposition: Yellow (ref)	–	–	–	–
Nurse disposition: Red-MD	1.10 (1.01—1.20)	1.09 (0.92—1.30)	1.078 (0.98—1.16)	0.89 (0.82—0.97)
Age: 21–40 years (ref)	–	–	–	–
Age: 0–1 years	0.86 (0.70—1.07)	1.17 (0.77—1.76)	1.19 (1.02—1.39)	1.09 (0.92—1.28)
Age: 2–5 years	0.97 (0.79—1.20)	1.24 (0.83—1.83)	0.89 (0.74—1.08)	1.48 (1.21—1.80)
Age: 6–20 years	0.96 (0.80—1.15)	0.83 (0.59—1.16)	0.95 (0.82—1.10)	1.49 (1.25—1.77)
Age: 41–65 years	0.90 (0.80—1.02)	1.13 (0.92—1.40)	1.09 (0.99—1.20)	0.89 (0.80—0.98)
Age: > 65 years	1.04 (0.91—1.19)	1.48 (1.16—1.88)	1.30 (1.17—1.45)	0.73 (0.64—0.83)
Sex: Female (ref)	–	–	–	–
Sex: Male	1.04 (0.94—1.14)	0.91 (0.76—1.08)	0.95 (0.88—1.03)	0.93 (0.85—1.01)
Economic dependency: 1 (least deprived) (ref)	–	–	–	–
Economic dependency: 2	1.25 (1.10—1.42)	1.35 (1.06—1.70)	1.05 (0.94—1.16)	0.89 (0.80 -1.00)
Economic dependency: 3	1.07 (0.93—1.23)	1.08 (0.84—1.40)	1.09 (0.98—1.22)	0.93 (0.83—1.05)
Economic dependency: 4	0.92 (0.80—1.06)	1.32 (1.03—1.69)	0.99 (0.89—1.11)	0.96 (0.85—1.09)
Economic dependency: 5 (most deprived)	0.89 (0.77—1.03)	1.08 (0.83—1.41)	1.09 (0.97—1.22)	0.93 (0.81—1.07)
Health Authority: Vancouver Coastal (ref)	–	–	–	–
Health Authority: Fraser	1.16 (1.03—1.30)	1.11 (0.89—1.39)	1.05 (0.96—1.15)	0.98 (0.89—1.09)
Health Authority: Interior	0.86 (0.73—1.00)	0.87 (0.65—1.16)	0.79 (0.70—0.89)	1.25 (1.08—1.44)
Health Authority: Vancouver Island	1.39 (1.21—1.59)	1.12 (0.88—1.43)	0.81 (0.72—0.91)	1.16 (1.02—1.33)
Health Authority: Northern	0.46 (0.35—0.61)	1.49 (1.02—2.18)	0.71 (0.59—0.85)	1.30 (1.04—1.63)
Rurality: Urban (ref)	–	–	–	–
Rurality: Rural	0.53 (0.46—0.61)	1.28 (1.01—1.62)	1.23 (1.11—1.37)	0.98 (0.87—1.12)
Health concern: Cardiovascular (ref)	–	–	–	–
Health concern: Dental/Mouth	0.90 (0.55—1.46)	0.68 (0.26—1.79)	0.89 (0.64—1.25)	1.53 (0.96—2.43)
Health concern: Dermatology	0.88 (0.68—1.14)	1.30 (0.82—2.05)	1.14 (0.93—1.39)	1.05 (0.82—1.35)
Health concern: First Aid	0.96 (0.70—1.31)	0.99 (0.54—1.80)	0.93 (0.71—1.22)	1.43 (1.07—1.92)
Health concern: Gastroenterology	0.94 (0.80—1.14)	0.93 (0.59—1.47)	0.96 (0.80—1.16)	0.87 (0.68—1.10)
Health concern: Gynecology	0.74 (0.56—0.98)	0.95 (0.55—1.63)	0.97 (0.78—1.21)	1.02 (0.73—1.42)
Health concern: Immunology	0.85 (0.62—1.17)	0.96 (0.49—1.88)	1.10 (0.84—1.44)	0.85 (0.64—1.12)
Health concern: Musculoskeletal	0.92 (0.75—1.13)	1.02 (0.66—1.58)	1.01 (0.84—1.22)	1.20 (0.94—1.53)
Health concern: Neurology	0.98 (0.80—1.20)	1.32 (0.84—2.06)	0.912 (0.75—1.12)	1.13 (0.88—1.46)
Health concern: Obstetrics/Postpartum	0.62 (0.46—0.83)	1.58 (0.91—2.75)	1.56 (1.23—1.97)	0.40 (0.30—0.53)
Health concern: Ophthalmology	1.05 (0.80—1.37)	0.70 (0.38—1.29)	1.03 (0.78—1.36)	1.31 (0.95—1.81)
Health concern: Other	0.78 (0.51—1.20)	1.28 (0.45—3.69)	1.09 (0.81—1.46)	0.96 (0.68—1.35
Health concern: Otolaryngology	1.05 (0.76—1.46)	1.27 (0.77—2.09)	1.19 (0.96—1.48)	0.91 (0.68—1.22)
Health concern: Pediatrics	0.90 (0.65—1.24)	1.43 (0.76—2.72)	0.92 (0.68—1.25)	0.74 (0.54—1.02)
Health concern: Pharmaceutical (Medication)	0.89 (0.28—2.81)	0.59 (0.14—2.51)	1.60 (1.03—2.46)	0.81 (0.50—1.31)
Health concern: Psychology (Mental Health)	0.71 (0.38—1.31)	1.20 (0.46—3.18)	0.88 (0.59—1.32)	0.53 (0.30—0.93)
Health concern: Respiratory	0.86 (0.70—1.07)	0.94 (0.58—1.51)	0.96 (0.78—1.18)	0.75 (0.59—0.96)
Health concern: Urology	1.02 (0.79—1.32)	1.38 (0.82—2.30)	1.17 (0.92—1.48)	0.77 (0.56—1.06)
Health concern: Wellness	0.78 (0.32—1.91)	0.812 (0.28—2.37)	1.60 (0.95—2.68)	1.49 (0.83—2.69)
Attached: Yes (ref)	–	–	–	–
Attached: No	1.01 (0.89—1.15)	0.92 (0.73—1.16)	0.76 (0.68—0.85)	1.22 (1.08—1.37)
Weekend: No (ref)	–	–	–	–
Weekend: Yes	1.05 (0.96—1.15),	0.93 (0.78—1.10),	0.93 (0.86—1.00)	0.97 (0.89—1.06)
Time of call: Day (ref)	–	–	–	–
Time of call: Evening	1.20 (1.09—1.31)	0.82 (0.65—1.04)	1.04 (0.97—1.12)	0.85 (0.79—0.93)
Time of call: Off-hours	1.02 (0.82—1.28)	1.13 (0.82—1.56)	1.01 (0.81—1.25)	1.19 (0.96—1.48)

^*1*^“Self-Manage” hazard ratios are inversely calculated, i.e., so that higher HRs for all dispositions indicate callers being more likely to be concordant

^*^Significant at alpha < 0.05

^**^Significant at alpha < 0.01

^***^Significant at alpha < 0.001

Callers in the “Go to the ED Now” disposition were more likely to be concordant with advice if they placed their call to 8–1-1 in the evening (1.20 [1.09–1.31]), or if their call was triaged by a nurse as “red-MD” rather than “yellow” (1.10 [1.01–1.20]). Individuals calling with an Obstetric/Postpartum health concern (0.62 [0.46 – 0.83]) or with a Gynecological concern (0.74 [0.56–0.98]) were less likely than those with a cardiovascular concern to be concordant.

Callers over 65 years old were more likely (1.48 [1.16–1.88]) to be concordant with physician advice to “Seek FP care now” compared with individuals in the 21–40 age category. Those at the second lowest level of economic deprivation (1.35 [1.06—1.70]) and second highest level (1.32 [1.03—1.69]), as compared to the least deprived, were more likely to be concordant. We observed no difference in concordance by the nature of the callers’ health concern for this disposition.

As with the “Seek FP care now” disposition, callers over 65 (1.30 [1.17–1.45]) and infants (1.19 [1.02–1.39]) advised “Schedule FP Appt” were more likely to be concordant than the 21 to40 year old reference category. Also, those with Obstetric /Postpartum (1.56 [1.23- 1.97]) or Pharmaceutical (Medication) (1.60 [1.03–2.46]) concerns were more likely to be concordant. Callers in this category without an attached FP were less likely to be concordant (0.76 [0.68–0.85]).

For the “Self-manage” disposition, there was a varying relationship with age, with children aged 2 to 5 (1.48 [1.21–1.80]) and children/adolescents 6 to 20 years old (1.49 [1.25–1.77]) more likely than the 21–40 reference category to stay home. Older adults (0.89 [0.80–0.98]) and seniors (0.73 [0.64–0.83]) were comparatively less likely to do so. Callers without an attached FP were more likely to be concordant (1.22 [1.08–1.37]), as were those whose medical concerns were in the “First Aid” category (1.43 [1.07–1.92]). In contrast, callers with health concerns in the Obstetrics/Postpartum (0.40 (0.30—0.53)], Psychology (MH) (0.53 [0.30 – 0.93]), and Respiratory (0.75 [0.59 – 0.96]) categories were less likely to be concordant. Those callers triaged to “RED” by a Nurse (vs yellow) (0.89 [0.82–0.97]), calling in the evening (vs day) (0.85 [0.79–0.93]) and at the second lowest level of economic dependency (vs lowest) (0.89 [0.80–1.0]) are also less likely.

We observed no significant difference in concordance by sex or weekends vs weekdays across any of the four dispositions (Table 6).

### Sensitivity analysis results

With expanded definitions of concordance that consider broader health service utilizations, we found higher rates of concordance over 48 h and seven days for the “Go to ED Now”, “Seek FP Care Now”, and “Schedule FP Appointment” dispositions. For the latter seven-day dispositions, we found a marked increase in events during the first two days, mainly due to the inclusion of ED visits. For the “Go to ED Now” disposition, there were increases at 12 and 24 h with the inclusion of physician encounters outside of the ED. We observed some additional small changes to the significance of independent variables in some models, but no changes in the direction of observed relationships (see Additional File 1: Table S3).

## Discussion

Few studies have examined the impact of the inclusion of virtual physicians in a telephone triage services on subsequent health service use [24] and these did not examine the concordance between physician advice and subsequent care seeking.

We found variable rates of concordance across the four dispositions “Go to ED Now”, “See FP now”, “Schedule FP Appointment”, and “Self-manage”. Concordance was highest for individuals instructed to go to an ED (70% concordant) and lowest for those instructed to seek care in their community (41% and 47%). The rate of concordance with advice to seek ED care we found was consistent with or higher than those reported in previous studies of nurse-led telephone triage services that do not include virtual physicians – which suggests VPs may reinforce urgency. However, rates of concordance with advice to seek the care of a FP was low compared with previous studies [7–9].

We found a significant effect of geography in all four models, with individuals living in rural areas (or in Northern Health Authority, which is predominantly rural and remote) having a fundamentally different pattern of service use than urban areas, with ED use being lower and FP use higher. This may be because rural FPs in BC may be more likely to provide urgent after hours care and visits outside of office compared to their urban counterparts [33]. Evidence from Alberta suggests that Family Medicine graduates who receive a rural as opposed to urban training program tend to have a broader scope of practice overall and urban-trained graduates who practice in rural areas tend to behave similarly, which supports the notion that rural care is typically more generalist and flexible [34]. This variation may also be driven at least in part by a substitution effect due to lack of accessible EDs and rural providers serving as both family and emergency physicians. Further research examining both distance from a callers’ home to the nearest ED and the difficulty of the route (e.g., mountainous regions, islands) may also reveal further trends, as we note that 90% of those concordant with advice to “Go to ED Now” did so within 4 h or less – suggesting relative proximity to an ED.

Current literature on the general population level impact of rurality on ED use has mostly focussed on lower-acuity presentations and signals are not always clear, however they suggest that lower SES and rurality are associated with higher ED use [12, 13], which is not evident in our results. Importantly, when we included visits to a FP in our concordance definition for our sensitivity analysis, we observed only marginal improvement in concordance by rurality. It is worth nothing that the impact of rurality on ED utilization appears to be driven at least in part by characteristics and health needs of the population under study [35–37]. As this study examines the general population, signals regarding the impact of rurality are likely to be mixed and would require examination of specific cohorts to quantify and address. It is also worth noting that “RED/YELLOW” HEiDi callers, particularly those told to “Go to ED Now”, are being assessed as likely *high* acuity, and so comparisons to the literature are not exact.

Concordance with advice to “Seek FP care now” was below 50%, and there was little difference in concordance between the “Seek FP care now” and “Schedule FP Appointment” dispositions. Primary care access challenges are pervasive and persistent in BC [15, 33, 38] and it is likely that this lack of access is driving at least some of the poor concordance. A 2022 qualitative study of patients seeking primary care attachment by Marshall et al. describes unattached patients as experiencing additional burdens due to lost opportunities for care and having to self-manage navigation of their health information and system resources [39]. They further note that these burdens are often worse for at-risk groups. Browne et al. examined access to primary care from an Indigenous perspective at an urban ED and found that ED use should be understood as “a function of patient’s prior and ongoing healthcare experiences in other settings” [14].

Lavergne et al.’s study on declining access to primary care in BC noted that within urban settings, encounters declined more rapidly in low-income areas while ED visits increased more rapidly. In this period, the percentage of physicians providing support for neighborhoods in the two lowest income quintiles declined from 30.6% to 26.3% [15]. Hedden et al. found a similar erosion of primary care in BC from 2006 to 2012, with the proportions of physicians providing care in alternative locations or after hours declined significantly across all practices in the range of 5–22% [33]. However, they also noted that rural physicians were significantly more likely to provide alternative locations or hours [33].

Concerningly, lack of attachment to a FP does not have a statistically significant effect on concordance for the most urgent FP advice category. This suggests that having a regular FP does not guarantee timely access to primary care, at least within the 7-day follow-up period we used. Additionally, if it is infeasible for patients to access an FP in the timely manner implied by the “Seek FP care now” disposition, we would suggest this advice category be modified, and possibly combined, with “Schedule FP Appointment” to reflect the real-world access to care of callers.

We did not observe differences in concordance by health concern, with the exception of callers with obstetric/postpartum and gynecological issues. Such patients appear to be less likely to attend an ED, more likely to schedule an FP appointment, and less likely to stay home when advised, suggesting that this group of patients may require additional communication efforts by VPs when provided advice and perhaps additional care pathways.

When conceptualizing potential strategies for HEiDi and Healthlink moving forward, we find some suggestions in the telehealth literature. In a 2016 systematic review of telehealth strategies in rural and remote Australia, Bradford et al. identified 6 key factors influencing success and sustainability: vision, ownership, adaptability, economics, efficiency, and equipment [40]. “Adaptability”, which the authors define as a strategy to “trial and model the service according to the needs of patients and health service” and “remain[ing] responsive to requirements of all stakeholders” [40], seems to be the most salient to the current BC context. Our results suggest that, while concordance rates on advice for ED use are high in aggregate, more work needs to be done to identify and address the needs of specific groups – particularly rural BC residents. Furthermore, advice to seek care from a family physician does not appear to be functioning as intended and may need to be recontextualized based on current access issues.

A final consideration for how callers’ may consider their health service use in BC, but arguably throughout Canada, should be the 2020 “In Plain Sight” report on Indigenous-specific racism and discrimination in healthcare. While out of scope of for this population-level analysis, the report highlights concerns around the perception of EDs and/or hospitals as unsafe may deter Indigenous persons from seeking care [41]. Similar perceptions may be shared by other racialized or historically marginalized groups and this should be examined by any future patient-centered work. Overall, it should be noted that ED utilization is driven at least in part by social determinants of health and should be considered using an equity lens.

### Limitations

This is a hypothesis-generating analysis using routinely collected provincial administrative data and is subject to all the limitations thereof including missing or incorrect data. We conducted this analysis during earlier phases of the COVID-19 pandemic when there were significant concerns about seeking in-person care, and this may have reduced ED and in-person physician visits; patients with access to virtual primary care (and this might include urban or wealthier patients) may have been more concordant.

It merits consideration that health categories may be miscategorized due to overlapping symptomology or miscommunication. Furthermore, it is not possible to determine whether the reason for a HEiDi call is the same reason for which a patient ultimately seeks in-person care as the HEiDi/811 service is not able to clinically determine and capture a standardized diagnostic code.

Furthermore, we did not have access to unique identifiers for nurse and/or VP providers in our data. This precluded the identification of baselines and trends within and across providers, including analyses such as clustering.

## Conclusion

Seventy percent of callers to a virtual physician triage service who are advised to go to an emergency department do so within 48-h, suggesting an overall high-degree of concordance with this advice. However, this is markedly lower in rural areas and the Northern health authority, which suggests the need for targeted assessment of access to emergency care, particularly with regard to distance and difficulty travelling from home to ED.

Less than half of those advised to seek follow-up care with a physician in the community do so within seven days, and there is little difference between those advised to seek FP care now and those advised to seek such care within seven days. In conjunction with recent literature, this may be driven by long-term trends restricting access to primary care in BC. Qualitative analysis of patient-caller’s decision making from their perspective would be beneficial in further understanding these findings. The drivers for the low rates of concordance with advice, particularly with advice to seek care in the community, merit future research.

### Supplementary Information

**Additional file 1: Supplementary Material.**

## Data Availability

**Table:** 

Dataset	Use of Data	Publisher(s)	Access Request Available	Public Links
Discharge Abstract Database (DAD)	Identification of hospitalization (inpatient acute care) events	BC Health Data Platform, Population Data BC	Yes	https://assets-hdp.healthbc.org/Dataset/details/2062e5b1-95fa-4a8a-98e9-68c9056e9ccf
				https://www.popdata.bc.ca/data/health/dad
National Ambulatory Care Reporting System (NACRS)	Identification of emergency department visits	BC Health Data Platform, Population Data BC	Yes	https://assets-hdp.healthbc.org/Dataset/details/d69f3377-6196-42e0-9f41-b303fd3ec4ae
				https://www.popdata.bc.ca/data/health/nacrs
Medical Services Plan (MSP) Billing	Identification of outpatient fee-for-service events	BC Health Data Platform, Population Data BC	Yes	https://assets-hdp.healthbc.org/Dataset/details/3fd6dd99-f027-4369-ba85-7aaa7388c58d
				https://www.popdata.bc.ca/data/health/msp
Medical Services Plan Client Registry	Confirmation of BC residency/enrolment in MSP/basic demographic information	BC Health Data Platform, Population Data BC	Yes	https://assets-hdp.healthbc.org/Dataset/details/124c5a97-c0e9-4397-98a7-600ec4f126ca
				https://www.popdata.bc.ca/data/demographic/consolidation_file
Vital Statistics: Deaths	Identifying mortality	BC Health Data Platform, Population Data BC	Yes	https://assets-hdp.healthbc.org/Dataset/details/26709839-2d3f-4250-b299-58975e3467a0
				https://www.popdata.bc.ca/data/demographic/vs_deaths
HealthLink BC Knowledge and Decision Record	Identifying HEiDi callers and their characteristics	HealthLink BC	No	(subset of data) https://assets-hdp.healthbc.org/Dataset/details/80844660-2c99-4c16-82d0-0c89e1333d19
Canadian Index of Multiple Deprivation (CIMD)	Socioeconomic status indicator from Statistics Canada	BC Health Data Platform	Yes	https://assets-hdp.healthbc.org/Dataset/details/acd22e65-1b7d-45e6-869e-ee182402b856 BC Health Data Platform, Population Data BC BC Health Data Platform, Population Data BC BC Health Data Platform, Population Data BC BC Health Data Platform, Population Data BC BC Health Data Platform, Population Data BC

## Abbreviations

ADI: Area Deprivation Index
BC: British Columbia
DAD: Discharge Abstract Database
ED: Emergency department
EMN: Emergency Medicine Network
FP: Family physician
HA: Health Authority
HDPBC: Health Data Platform BC
HEiDi: HealthLink BC Emergency iDoctors in-assistance
HLBC: HealthLink BC
MSP: Medial Services Plan
NACRS: National Ambulatory Care Reporting System
PHN: Personal Health Number
SES: Socioeconomic Status
TTE: Time-to-event
VP: Virtual Physician
