# Supplementary material for: Concordance between 8-1-1 HealthLink BC Emergency iDoctor-in-assistance (HEiDi) virtual physician advice and subsequent health service utilization for callers to a nurse-managed provincial health information telephone service
Source: BMC Health Serv Res. 2023 Sep 27;23:1031. doi: 10.1186/s12913-023-09821-w (PMC10523598; doi:10.1186/s12913-023-09821-w)
Supplement: Supplementary file 1 — Additional file 1: Supplementary Material. [file 12913_2023_9821_MOESM1_ESM.docx]

# Supplementary Material

## Health Concerns

| **Table S1. Callers’ health concerns by HEiDi disposition.** | | | | |
| --- | --- | --- | --- | --- |
|  | **HEiDi Disposition** | | | |
| **Health Concern** | **Go to ED Now**  **(n=2876, 16.7%)** | **Seek FP Care Now**  **(n=1512, 8.8%)** | **Schedule an FP Appointment**  **(n=6649, 38.7%)** | **Self-manage**  **(n=6151, 35.8%)** |
| Cardiovascular (Heart/Circulation) | 193 (6.7%) | 72 (4.8%) | 318 (4.8%) | 184 (3.0%) |
| Dental/Mouth | 27 (0.9%) | 17 (1.1%) | 115 (1.7%) | 89 (1.4%) |
| Dermatology (Skin, Hair, Nails) | 158 (5.5%) | 147 (9.7%) | 637 (9.6%) | 719 (11.7%) |
| Diabetes^1^ | 18 (0.6%) | ≤ 5 (≤ 0.3%) | 48 (0.7%) | 29 (0.5%) |
| Endocrinology (Glands)^1^ | ≤ 5 (≤ 0.2%) | ≤ 5 (≤ 0.3%) | 22 (0.3%) | 13 (0.2%) |
| First Aid | 85 (3.0%) | 52 (3.4%) | 191 (2.9%) | 368 (6.0%) |
| Gastroenterology (Digestive) | 583 (20.3%) | 208 (13.8%) | 1078 (16.2%) | 1012 (16.5%) |
| Gynecology (Women’s Reproductive) | 127 (4.4%) | 83 (5.5%) | 419 (6.3%) | 181 (2.9%) |
| Hematology (Blood)^1^ | ≤ 5 (≤ 0.2%) | 0 | ≤ 5 (≤ 0.1%) | ≤ 5 (≤ 0.1%) |
| Immunology | 71 (2.5%) | 34 (2.2%) | 171 (2.6%) | 268 (4.4%) |
| Infectious/Communicable Diseases^1^ | 13 (0.5%) | ≤ 5 (≤ 0.3%) | 50 (0.8%) | 74 (1.2%) |
| Musculoskeletal (Bone, Muscle, Joint) | 376 (13.1%) | 241 (15.9%) | 894 (13.4%) | 802 (13.0%) |
| Neurology | 362 (12.6%) | 160 (10.6%) | 684 (10.3%) | 683 (11.1%) |
| Obstetrics & Postpartum | 115 (4.0%) | 51 (3.4%) | 253 (3.8%) | 178 (2.9%) |
| Oncology (Site-Specific Cancers)^1^ | ≤ 5 (≤ 0.2%) | ≤ 5 (≤ 0.3%) | 6 (0.1%) | ≤ 5 (≤ 0.1%) |
| Ophthalmology (Eyes) | 115 (4.0%) | 55 (3.6%) | 168 (2.5%) | 224 (3.6%) |
| Otolaryngology (Ear, Nose, Throat) | 75 (2.6%) | 90 (6.0%) | 382 (5.7%) | 262 (4.3%) |
| Pediatrics | 88 (3.1%) | 43 (2.8%) | 180 (2.7%) | 230 (3.7%) |
| Pharmaceutical (Medication) | ≤ 5 (≤ 0.2%) | 7 (0.5%) | 37 (0.6%) | 44 (0.7%) |
| Psychology (Mental Health) | 15 (0.5%) | 10 (0.7%) | 74 (1.1%) | 23 (0.4%) |
| Respiratory | 304 (10.6%) | 140 (9.3%) | 604 (9.1%) | 569 (9.3%) |
| Urology (Urinary Tract & Male Genitalia) | 135 (4.7%) | 81 (5.4%) | 291 (4.4%) | 147 (2.4%) |
| Wellness | 6 (0.2%) | 10 (0.7%) | 24 (0.4%) | 44 (0.7%) |
| *Note: small cell numbers (1 ≤ n ≤ 5) are censored as “≤ 5” to minimize risk of re-identification.*  *^1^Due to the small number of cases in at least one of the dispositions, these five concerns were collapsed into an “Other” category for the TTE analysis to ensure model convergence.* | | | | |

## Additional TTE Results

| **Table S2. Cumulative number of “events” (i.e., recorded VP-advised health service encounters) by HEiDi disposition over 48 hours or 7 days.** | | | | |
| --- | --- | --- | --- | --- |
| **Time-to-Event (hours or days)** | **Go to ED Now (n=2876)** | **Seek FP Care Now (n=1512)** | **Schedule an FP Appointment (n=6649)** | **Self-manage^1^ (n=6151)** |
| **0** | 384 (13.4%) | 261 (17.3%) | 459 (6.9%) | 422 (6.9%) |
| **1** | 1441 (50.1%) | 418 (27.6%) | 1545 (23.2%) | 1069 (17.4%) |
| **2** | 1715 (59.6%) | 493 (32.6%) | 2045 (30.8%) | 1480 (24.1%) |
| **3** | 1798 (62.5%) | 538 (35.6%) | 2367 (35.6%) | 1805 (29.3%) |
| **4** | 1840 (64.0%) | 570 (37.7%) | 2602 (39.1%) | 2035 (33.1%) |
| **5** | 1859 (64.6%) | 594 (39.3%) | 2810 (42.3%) | 2204 (35.8%) |
| **6** | 1867 (64.9%) | 611 (40.4%) | 2962 (44.5%) | 2353 (38.3%) |
| **7** | 1872 (65.1%) | 625 (41.3%) | 3091 (46.5%) | 2471 (40.2%) |
| **12** | 1917 (66.7%) | - | - | - |
| **18** | 1963 (68.3%) | - | - | - |
| **24** | 1996 (69.4%) | - | - | - |
| **30** | 2008 (69.8%) | - | - | - |
| **36** | 2010 (69.9%) | - | - | - |
| **42** | 2017 (70.1%) | - | - | - |
| **48** | 2025 (70.4%) | - | - | - |
| *Note: The “Go to ED Now” time-to-event values indicate hourly intervals and the other dispositions indicate daily intervals. Thus, “0” indicates an event being recorded in within the first half-hour or on the same calendar day as the HEiDi call.*  *^1^The “Home Treatment” disposition is inverted, so the increasing incidence of events indicates discordance.* | | | | |

**Figure S1. Adjusted survival curves stratified by Health Authority for the “Go to ED Now” disposition.**

****Additional Sensitivity Analysis Results

**Figure S2. Adjusted Kaplan-Meier survival curves for each VP advice category from the sensitivity analysis.**

| **Table S3. Sensitivity summary of caller and call characteristics impact on concordance by category of advice (adjusted hazard ratios of multivariate cox regression)** | | | |
| --- | --- | --- | --- |
| **Variable** | **Go to ED now**  **(n_events_ = 2492)** | **Seek FP Care Now**  **(n_events_ = 1183)** | **Schedule an FP Appointment**  **(n_events_ = 4702)** |
| **Likelihood ratio test (X^2^ [df] p-value)** | 145.2 (38) p < 0.001 | 59.9 (38) p =0.01 | 211.6 (38) p < 0.001 |
| **Nurse disposition: Yellow (ref)** | -- | -- | -- |
| **Nurse disposition: Red-MD** | 1.046 (0.965 - 1.134) | 1.194 (1.056 - 1.351) | 1.067 (0.998 - 1.141) |
| **Age: 21-40 years (ref)** | -- | -- | -- |
| **Age: 0-1 years** | 0.905 (0.746 - 1.097) | 1.164 (0.867 - 1.562) | 0.907 (0.796 - 1.033) |
| **Age: 2-5 years** | 1.028 (0.846 - 1.25) | 1.342 (1.021 - 1.764) | 0.861 (0.742 - 1) |
| **Age: 6-20 years** | 1.037 (0.883 - 1.218) | 1.082 (0.864 - 1.355) | 0.976 (0.869 - 1.096) |
| **Age: 41-65 years** | 0.985 (0.882 - 1.1) | 1.118 (0.96 - 1.303) | 1.003 (0.929 - 1.084) |
| **Age: >65 years** | 1.078 (0.956 - 1.215) | 1.253 (1.044 - 1.504) | 1.158 (1.058 - 1.266) |
| **Sex: Female (ref)** | -- | -- | -- |
| **Sex: Male** | 1.021 (0.936 - 1.113) | 0.87 (0.764 - 0.99) | 0.939 (0.881 - 1.001) |
| **Economic dependency: 1 (least deprived) (ref)** | -- | -- | -- |
| **Economic dependency: 2** | 1.161 (1.032 - 1.306) | 1.158 (0.977 - 1.372) | 1.058 (0.972 - 1.153) |
| **Economic dependency: 3** | 0.993 (0.875 - 1.127) | 1.056 (0.884 - 1.261) | 1.053 (0.964 - 1.149) |
| **Economic dependency: 4** | 0.911 (0.803 - 1.034) | 1.063 (0.887 - 1.275) | 1.003 (0.916 - 1.098) |
| **Economic dependency: 5 (most deprived)** | 0.897 (0.789 - 1.021) | 1.049 (0.864 - 1.273) | 1.012 (0.919 - 1.114) |
| **Health Authority: Vancouver Coastal (ref)** | -- | -- | -- |
| **Health Authority: Fraser** | 1.092 (0.98 - 1.218) | 1.086 (0.928 - 1.27) | 0.988 (0.914 - 1.068) |
| **Health Authority: Interior** | 0.961 (0.841 - 1.097) | 0.982 (0.801 - 1.204) | 0.911 (0.825 - 1.005) |
| **Health Authority: Vancouver Island** | 1.254 (1.103 - 1.425) | 1.028 (0.861 - 1.229) | 0.887 (0.81 - 0.971) |
| **Health Authority: Northern** | 0.671 (0.55 - 0.819) | 0.997 (0.736 - 1.351) | 0.76 (0.656 - 0.88) |
| **Rurality: Urban (ref)** | -- | -- | -- |
| **Rurality: Rural** | 0.74 (0.66 - 0.83) | 1.054 (0.878 - 1.264) | 1.069 (0.98 - 1.166) |
| **Health concern: Cardiovascular (ref)** | -- | -- | -- |
| **Health concern: Dental/Mouth** | 0.838 (0.529 - 1.329) | 0.617 (0.311 - 1.225) | 0.8 (0.608 - 1.052) |
| **Health concern: Dermatology** | 0.9 (0.713 - 1.135) | 0.982 (0.706 - 1.366) | 0.962 (0.815 - 1.136) |
| **Health concern: First Aid** | 0.987 (0.745 - 1.308) | 0.858 (0.562 - 1.309) | 0.739 (0.586 - 0.932) |
| **Health concern: Gastroenterology** | 0.961 (0.806 - 1.147) | 1.065 (0.78 - 1.454) | 0.962 (0.825 - 1.122) |
| **Health concern: Gynecology** | 0.86 (0.671 - 1.103) | 1.061 (0.734 - 1.533) | 0.929 (0.776 - 1.112) |
| **Health concern: Immunology** | 0.942 (0.702 - 1.262) | 1.254 (0.797 - 1.971) | 1.311 (1.055 - 1.629) |
| **Health concern: Musculoskeletal** | 0.927 (0.767 - 1.119) | 0.963 (0.712 - 1.304) | 0.978 (0.839 - 1.14) |
| **Health concern: Neurology** | 0.965 (0.798 - 1.167) | 1.069 (0.777 - 1.471) | 0.98 (0.835 - 1.151) |
| **Health concern: Obstetrics/Postpartum** | 0.834 (0.646 - 1.078) | 1.284 (0.85 - 1.94) | 1.383 (1.137 - 1.684) |
| **Health concern: Ophthalmology** | 1.084 (0.848 - 1.387) | 0.785 (0.516 - 1.193) | 1.085 (0.868 - 1.355) |
| **Health concern: Other** | 0.832 (0.569 - 1.217) | 1.41 (0.694 - 2.866) | 1.079 (0.847 - 1.374) |
| **Health concern: Otolaryngology** | 1.072 (0.802 - 1.433) | 1.287 (0.901 - 1.838) | 1.396 (1.17 - 1.666) |
| **Health concern: Pediatrics** | 0.943 (0.698 - 1.275) | 1.009 (0.627 - 1.624) | 1.116 (0.874 - 1.426) |
| **Health concern: Pharmaceutical (Medication)** | 0.731 (0.232 - 2.3) | 1.041 (0.413 - 2.623) | 1.147 (0.773 - 1.704) |
| **Health concern: Psychology (Mental Health)** | 0.625 (0.347 - 1.127) | 0.695 (0.328 - 1.476) | 0.81 (0.585 - 1.121) |
| **Health concern: Respiratory** | 0.846 (0.693 - 1.032) | 1.153 (0.831 - 1.598) | 1.415 (1.203 - 1.663) |
| **Health concern: Urology** | 1.106 (0.873 - 1.402) | 1.365 (0.952 - 1.958) | 1.353 (1.122 - 1.632) |
| **Health concern: Wellness** | 0.755 (0.309 - 1.843) | 0.531 (0.227 - 1.242) | 1.233 (0.771 - 1.972) |
| **Attached: Yes (ref)** | -- | -- | -- |
| **Attached: No** | 1.033 (0.922 - 1.156) | 0.915 (0.776 - 1.08) | 0.861 (0.793 - 0.936) |
| **Weekend: No (ref)** | -- | -- | -- |
| **Weekend: Yes** | 1.035 (0.95 - 1.127) | 0.854 (0.752 - 0.971) | 0.991 (0.932 - 1.055) |
| **Time of call: Day (ref)** | -- | -- | -- |
| **Time of call: Evening** | 1.083 (0.995 - 1.178) | 0.948 (0.81 - 1.109) | 0.981 (0.924 - 1.041) |
| **Time of call: Off-hours** | 1.013 (0.826 - 1.242) | 1.209 (0.944 - 1.55) | 1.056 (0.886 - 1.259) m |

| **Table S4. Cumulative number of “events” (i.e., recorded VP-advised health service encounters) by HEiDi disposition over 48 hours or 7 days following the sensitivity analysis definitions.** | | | |
| --- | --- | --- | --- |
| **Time-to-Event (hours or days)** | **Go to ED Now (n=2876)** | **Seek FP Care Now (n=1512)** | **Schedule an FP Appointment (n=6649)** |
| **0** | 384 (13.4%) | 669 (44.2%) | 1053 (15.8%) |
| **1** | 1441 (50.1%) | 936 (61.9%) | 2849 (42.8%) |
| **2** | 1715 (59.6%) | 1030 (68.1%) | 3547 (53.3%) |
| **3** | 1798 (62.5%) | 1078 (71.3%) | 3929 (59.1%) |
| **4** | 1840 (64.0%) | 1119 (74.0%) | 4186 (63.0%) |
| **5** | 1859 (64.6%) | 1145 (75.7%) | 4411 (66.3%) |
| **6** | 1867 (64.9%) | 1160 (76.7%) | 4570 (68.7%) |
| **7** | 1872 (65.1%) | 1183 (78.2%) | 4702 (70.7%) |
| **12** | 2225 (77.4%) | - | - |
| **18** | 2270 (78.9%) | - | - |
| **24** | 2412 (83.9%) | - | - |
| **30** | 2422 (84.2%) | - | - |
| **36** | 2424 (84.3%) | - | - |
| **42** | 2430 (84.5%) | - | - |
| **48** | 2492 (86.6%) | - | - |
| *Note: The “Go to ED Now” time-to-event values indicate hourly intervals and the other dispositions indicate daily intervals. Thus, “0” indicates an event being recorded in within the first half-hour or on the same calendar day as the HEiDi call.* | | | |
